# Bilateral external ear canal osteomas – discussion on a clinical case

**Published:** 2016

**Authors:** DC Gheorghe, AE Stanciu, A Ulici, A Zamfir-Chiru-Anton

**Affiliations:** * Department of Otorhinolaryngology, “M. S. Curie” Children Emergency Hospital, Bucharest, Romania; “Carol Davila” University of Medicine and Pharmacy, Bucharest, Romania; ** Oncology Institute Bucharest, Romania; *** Department of Otorhinolaryngology, “G. Alexandrescu” Children Emergency Hospital, Bucharest, Romania; **** Department of Orthopaedics, “G. Alexandrescu” Children Emergency Hospital, Bucharest, Romania

**Keywords:** osteoma, external ear canal, external ear cholesteatoma

## Abstract

Osteomas of the external ear are uncommon benign tumors that need to be differentiated from the external ear canal exostoses, bony proliferations that are linked mainly to cold-water exposure. Clinical manifestations vary from no symptoms to recurrent local infections and external ear cholesteatoma.

**Objective:** presenting a rare case that we did not find described in the published literature. A patient with multiple long-term asymptomatic osteomas of both external ear canals presented to our department.

**Material:** Data recorded from the patient’s medical record was reviewed and analyzed. Surgery was performed and histology confirmed the presumptive diagnosis.

**Results:** There was a discrepancy between the local severity of the disease, with a complete obstruction of his ear canals, and the long-term disease-free status of the patient.

**Conclusion:** We hypothesized about the etiology of these multiple bilateral osteomas of the EAC, in light of the clinical and surgical findings.

External ear canal obstruction is a relatively common condition due to various causes: cerumen impaction, poor epidermal clearance, local infection, local tumors, or foreign bodies. Bony growths in the external ear have been largely classified as exostoses (most frequently encountered) and osteomas, to differentiate their mechanisms of origin. Although the exact intrinsic mechanism that triggers their development is unknown, most of the authors agree that exostoses are a reaction to cold-water stimulation of the local periosteum[**1**], while osteomas are benign osseous tumors[**2**]. The treatment of both conditions is the surgical removal although the method is debatable[**3**]. 

The development of these osseous tumors is a long-term process. During this time, symptoms can develop and make the patient seek for medical care. An uncommon case was referred to our service, with an almost complete osseous obstruction of both his ear canals and no significant hearing loss for a long time. We presented this case herein and discussed its causes and the dilemmas that arose. 

## Case presentation

**Fig. 1:**
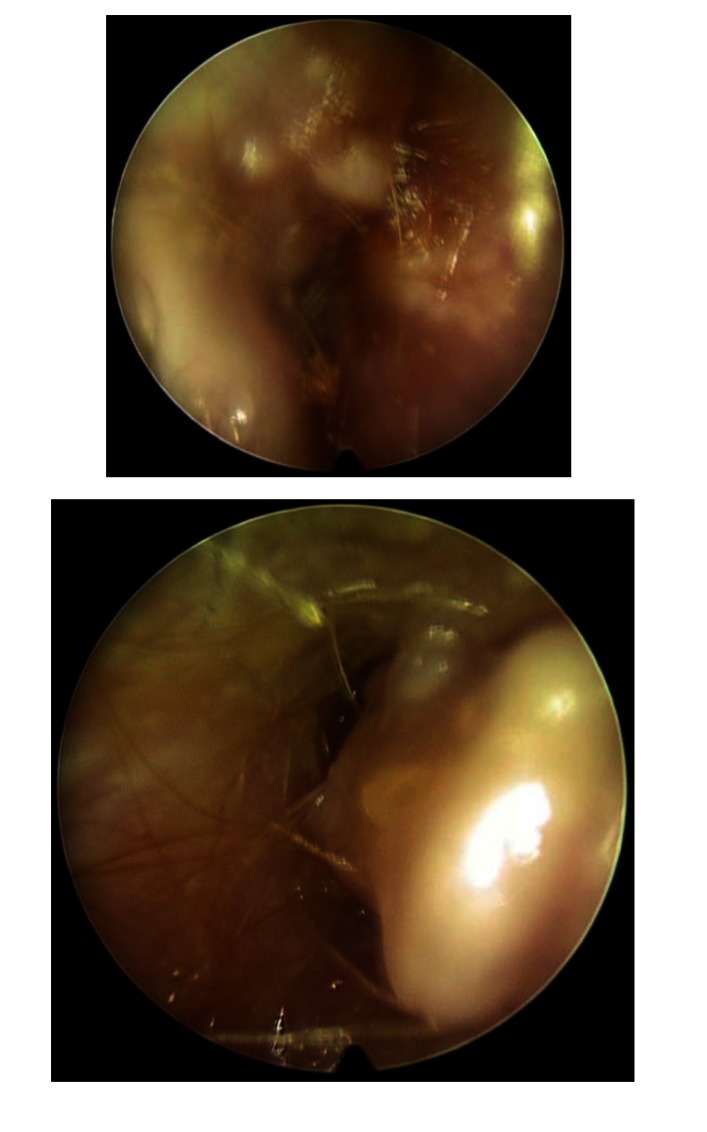
Multiple, irregular shaped osseous tumors in external ear canals: the left and the right side respectively; no space left for the examination of the eardrums

A 15-year-old boy was admitted to our department for short time hearing loss, of approximately 1-week duration. The clinical examination noted a very important obstruction of both ear canals. No ear specula could be used to examine the tympanic membrane. Practically, no lumen was visible/ accessible by otoscopy (**Fig. 1**). No history of prolonged water sports was recorded and no otologic complaints were present before the actual episode. No history of otitis could be elicited.

**Fig. 2:**
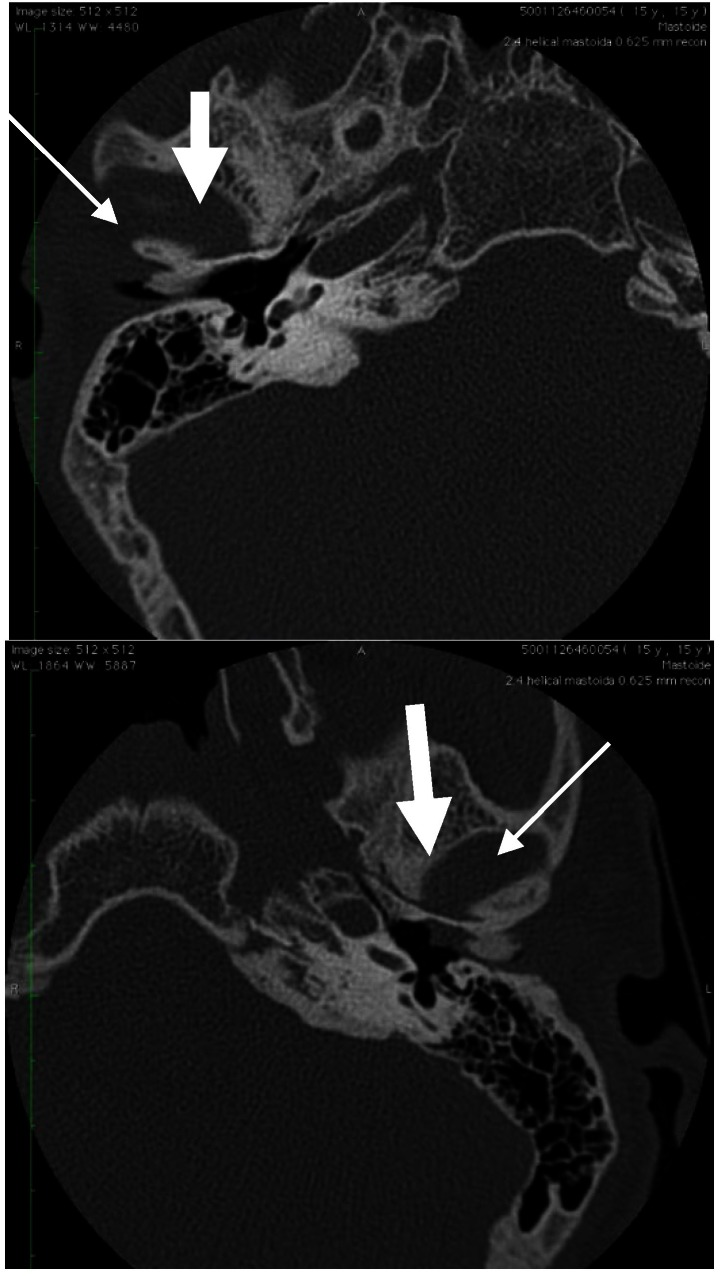
CT-scan revealing external ear osteomas on both sides, with insertion of the ear canal (thin arrows) on the anterior wall; the space outside TM on the right side is clear; the same space on the left ear is full (thick arrows)

A CT-scan was performed andnormal middle ear spaces were identified, on both sides, with clear visible eardrums. The external halves of the ear canals were obstructed by multiple osseous tumors, with relatively small insertion pedicles (compared to the tumors) and spiky, irregular surface. The internal part of the ear canals were: free on the right side and full with a soft mass on the left side (explaining the symptoms of our patient) (**Fig. 2**). 

**Fig. 3:**
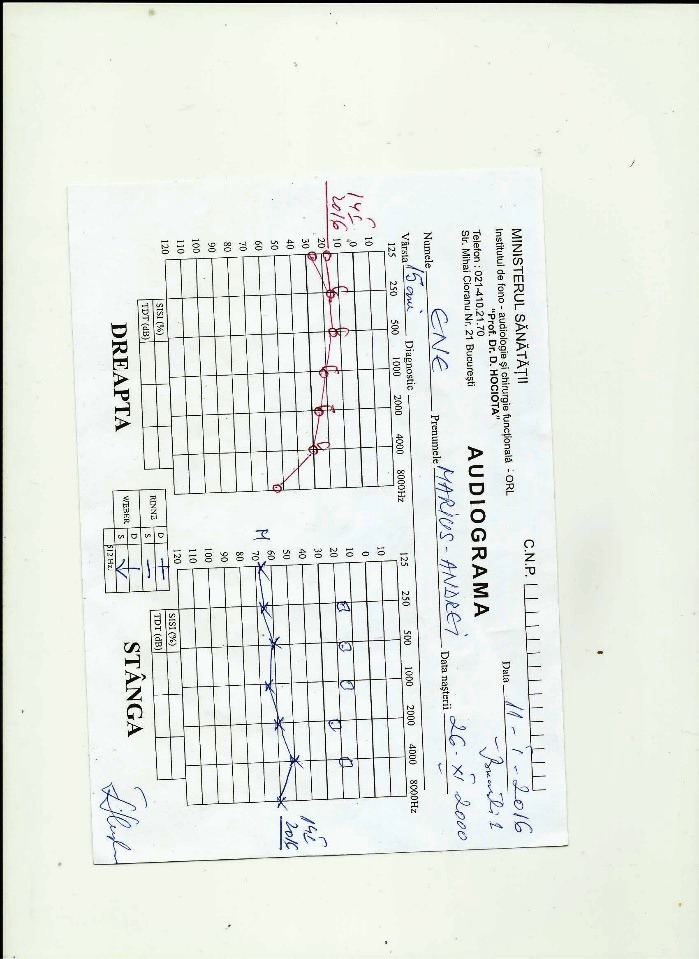
Patient’s audiogram

An audiogram revealed a pure conduction hearing loss on his left side (**Fig. 3**). 

Surgery was advised and performed on the left, symptomatic ear. The multiple osteomas found had large but steep pedicles with the origin into the middle third of the ear canal. They were removed by using diamond burrs of appropriate dimensions with the aid of an operating microscope, through a canal approach. Macerated epidermal debris and cerumen were found on the left eardrum, after osteoma removal. The tympanic membrane and the annulus were left intact. Some extracted bone fragments were sent for histological evaluation. The paraffin-embedded sections confirmed the presence of osteoma.

## Discussion

Osteomas are benign tumors and their incidence is cited in 0,05% of the otologic surgeries[**4**]. They can sometimes generate external ear cholesteatoma[**5**]. Their etiology is still unknown, although different possible causes have been discussed in the literature[**5**].

Our case was very unusual due to the discrepancy between the patient’s symptoms and the clinical CT examinations. The severe osseous obstruction of both his ear canals would have produced a bilateral conductive hearing loss, for a longer period than recorded. The age of the patient (15years) was suggestive of 2 possible explanations: either a very fast tumor growth or an extremely well tolerated congenital disorder. 

The first hypothesis could be proposed due to the bilaterality of the lesions. Exostoses could have been considered as a first diagnosis but the amount of bone obstruction, the lack of local constant water irritation, and the unusual CT appearance of the tumors granted the condition rather unlikely. 

The spiky and irregular shape of the tumors suggested a tumoral etiology. Still, bilaterality and a symptom free disease could give rise to suspicions as to a congenital unrecognized condition. Also, the multilocular origin of the osseous growths suggested un unusual pattern for a tumor, be it malignant or benign in nature. 

The only acceptable explanation for our patient’s disease was a congenital malformation, existent from birth. The lack of hearing loss for such a chronic disease is still debatable. The epidermal clearance mechanism of the ear canal was apparently functional, a thing that was obvious from the analysis of the CT scans of the symptom-free (right) ear. The left ear hearing thresholds were afflicted only when epidermal debris and cerumen accumulated behind the external osseous obstruction affected the tympanic membrane. 

One could assume that the bony growths of the patient enlarged progressively, followed eventually by a disturbance of canal aeration and epidermal clearance. 

The dilemmas that we found in this case were the real nature of the obstruction (exostosis vs. osteoma) and the mechanism of genesis (malformation vs. postnatal). We did a research of PubMed literature for similar reported cases, but we could not find any. Therefore, we considered the disease congenital bilateral osteomas of the ear canal. It would have been interesting to monitor the development of the patient’s tumors over time. A long-term follow-up is in progress with the purpose of surveying the possible recurrences in the operated ear. Another particular aspect of this case was its asymptomatic course until teenagehood. 

The surgical treatment of the EAC osteoma is mandatory in cases with significant morbidity: hearing loss, epidermal retention, or recurrent infections[**4**,**6**]. We also decided to operate the symptomatic ear. The technical approach varies according to the extent of the disease[**5**,**7**]. Our intra-auricular procedure seemed a good choice due to the lack of mastoid or middle ear involvement. Intraoperatively, we could confirm the macerated epidermal debris present outside the tympanic membrane. No trauma to the eardrum occurred.

A prolonged healing after surgery is mentioned in the literature if no split thickness grafts are used[**6**,**8**]. We did not consider their use, since much of the ear canal was carefully preserved during the procedure. Some authors stressed the risk of hearing loss due to trauma from surgical drilling[**6**]. We used only diamond burrs in our surgery, for maximal hearing preservation. 

The patient is still in follow-up for the monitoring of the local healing process and the eventual recurrences. 

## Conclusion

Osseous malformations of the external ear can take various histological presentations. The lack of epidermal clearance and aeration are always followed by complaints, going from hearing loss to local infection and otorrhea. Surgery is indicated if the disease becomes symptomatic. Local monitoring of the disease can appreciate the recurrence potential of the condition. It is very unusual for such a disease to go undiagnosed until later in life. 

**Contribution of authors**

All authors have equally contributed to this work.
